# Expired Cefalexin Loaded into Mesoporous Nanosilica for Self-Healing Epoxy Coating on 304 Stainless Steel

**DOI:** 10.3390/nano12142406

**Published:** 2022-07-14

**Authors:** Beibei Yang, Jiayu Dong, Haifeng Bian, Haimin Lu, Duan Bin, Shaochun Tang, Yaqiong Song, Hongbin Lu

**Affiliations:** 1 Department of Chemistry and Chemical Engineering, Nantong University, Nantong 226019, China; 17110220059@fudan.edu.cn; 2 Haian Institute of High-Tech Research, College of Engineering and Applied Science, Nanjing University, Nanjing 210033, China; njudjy12345@163.com (J.D.); luhm@nju.edu.cn (H.L.); 3 College of Engineering and Applied Sciences, Nanjing University, Nanjing 210033, China; bhfjsyz@163.com; 4 Jiangsu Guojiao New Material Co., Ltd., Rugao 226599, China; siyero@163.com

**Keywords:** self-healing coating, slow release, cefalexin, mesoporous silica, corrosion resistance

## Abstract

A self-healing epoxy coating is creatively prepared by employing expired cefalexin loaded into mesoporous silica nanomaterials (MSNs) for corrosion protection of 304 stainless steel (304SS). A series of physical characterizations, including transmission electron microscopy (TEM), Fourier transform infrared (FTIR) spectrometer, and N_2_ adsorption–desorption isotherms, verified that the cefalexin successfully filled porous MSN. The corrosion resistance of the epoxy (EP) coating incorporated with the cefalexin@MSNs is investigated using a Tafel polarization curve and electrochemical impedance spectra (EIS) in a 3.5 wt.% NaCl solution. It is found that the EP-Cefalexin@MSNs coating has a higher self-corrosion voltage and a lower self-corrosion current density than EP coating. Moreover, the charge transfer resistance (R_ct_) value of Cefalexin@MSNs coating is twice that of EP coating after immersion for 24 h, indicating that the cefalexin@MSNs significantly enhance the corrosion resistance of the coating under long-duration immersion. The improved corrosion resistance is attributed to the densified adsorption of the cefalexin inhibiting the cathode corrosion reaction, providing a self-healing long-duration corrosion protection for 304SS.

## 1. Introduction

Corrosion of stainless steel (SS) causes massive economic loss annually, and polymer coatings remain the most convenient and effective method for protecting SS from corrosion [1,2,3,4]. The polymer coatings provide physical barriers which isolate corrosive media from the SS substrate. However, when long-duration exposed in the corrosive media, defects such as pores and cracks appear easily in the polymer coatings, leading to the failure of protection [5]. Currently, self-healing polymer coating is becoming favorable, since it not only provides a physical barrier but also automatically repairs coating defects [6,7,8,9,10,11,12]. The content of corrosion inhibitor in organic coating can be increased by loading corrosion inhibitor into a nanocontainer, and corrosion inhibitor will be released from the nano container to achieve self-repair of the coating [13,14].

Mesoporous silica nanomaterials (MSNs) are good candidates for loading corrosion inhibitors due to their large specific surface area, controllable pore volume, strong loading capacity, high stability, and good compatibility [15]. The inhibitors loaded in MSNs greatly enhance the corrosion resistance of polymer coatings [16,17,18,19,20]. Borisova et al. dipped mesoporous silica loaded with 1H-benzotriazole (BTA) into sol–gel coating to protect aluminum alloys from corrosion and proved that the mixed coating improves anticorrosive performance [16]. Similarly, Falcòn et al. [21] reported the active corrosion protection of carbon steel by highly ordered mesoporous silica loaded with dodecylamine and confirmed that the prepared coating has good active corrosion protection during the corrosion process. Zea et al. [22] prepared homogeneous mesoporous silica nanoparticles and loaded an environmentally friendly corrosion inhibitor—sodium phosphomolybdate—into mesoporous silica nanoparticles, which greatly improved the anticorrosive performance of the coating. Recloux et al. [23] synthesized a thin mesoporous silica film through the evaporation-induced self-assembly process and doped with benzotriazole to achieve active corrosion protection. Therefore, modification of the coating defects and utilization of MSN are feasible methods of improving the corrosion performance of stainless steel.

The modern environment often faces the issues of accumulation of abandoned or expired drugs; it is well known that improper handling of expired drugs will lead to serious safety and environmental pollution, so these drugs should be handled reasonably. Recently, some date-expired drugs have been proved to be good inhibitors for mitigating corrosion, and the inhibitors came from organic compounds containing O, N, and S elements [24,25,26,27]. The cephalexin compounds contain π bonds, benzene rings, conjugated double bonds, and heteroatoms (O, N, and S) that make them as corrosion inhibitors for steel in an acid medium [28,29]. This distinguished feature is the motivation worldwide for investigating the potential of expired organic drugs as corrosion inhibitors. However, the composite corrosion-resistant coating based on the adsorption of mesoporous materials and organic drugs such as cephalexin as inhibitors have never been reported.

In the present work, the expired drug cefalexin is used as a corrosion inhibitor to reduce the social and environmental hazards of expired drugs. The MSN-loaded cefalexin is expected to achieve a slow release of corrosion inhibitor, thereby prolonging the anticorrosion effect of the coating. A self-healing coating was prepared by using the expired cefalexin as a corrosion inhibitor, the MSNs as encapsulation materials, and epoxy as a binder. The microstructure and the properties of the Cefalexin@MSNs were characterized by transmission electron microscopy (TEM), N_2_ adsorption–desorption isotherms, and Fourier transform infrared spectroscopy (FTIR). Ultraviolet–visible spectroscopy was used to detect the sustained release properties of the Cefalexin@MSNs. The corrosion resistance of the epoxy coating incorporated with the Cefalexin@MSNs was studied using a Tafel polarization curve and electrochemical impedance spectra (EIS) in 3.5 wt.% NaCl solution, and the self-healing mechanism was also investigated.

## 2. Materials and Methods

### 2.1. Materials and Instruments

The materials include cefalexin (LGM Pharma Co. Ltd., Colorado Springs, CO, USA, beyond the expiration date by 30 days), tri-copolymer poly(ethylene glycol)-block-poly(propylene glycol)-block-poly(ethylene glycol), (P123, EG_20_PG_40_EG_20_, average molecular weight 5800, Shanghai Macklin Biochemical Co. Ltd., Shanghai, China), tetraethyl orthosilicate (TEOS, Shanghai Chemical Leechdom Co. Ltd., Shanghai, China), sodium chloride (NaCl) (Aladdin Reagent Co. Ltd., Shanghai, China), and hydrochloric acid (HCl) (Beijing Chemical Industry Group Plant Co. Ltd., Beijing, China). The 304SS was provided by Haimeng Senda Decoration Materials Co. Ltd., Nantong, China, and its composition is presented in Table 1.

### 2.2. Synthesis of MSNs

4.0 g of P123 was dissolved in 120 mL HCl solution (3 mol·L^−^^1^) under continuous stirring at 40 °C for 2 h, then 8.5 g of TEOS was added under stirring for 24 h. Then, the obtained suspension was transferred into a Teflon-lined stainless steel autoclave and heated at 100 °C for 24 h. The obtained product was separated by decompression suction filtration and rinsed with ethanol and deionized water, then dried at 40 °C in a vacuum oven and calcined at 550 °C in a tube furnace to remove the P123.

### 2.3. Preparation of Cefalexin@MSNs and EP-Cefalexin@MSNs Coatings

Amounts of 0.2 g of cephalexin and 40 mg of MSNs were dissolved in 40 mL of DI water and stirred for 48 h to reach adsorption equilibrium, then naturally dried at room temperature to obtain Cefalexin@MSNs. The 2 wt.% Cefalexin@MSNs was dispersed into the epoxy (EP) under ball-milling with zirconium beads for 4 h to obtain the EP-Cefalexin@MSNs coating. The 304SS substrates (25 mm × 25 mm × 1 mm) were polished by the sandpapers with different particle sizes of 400, 600, 1200, 1500, and 2000 mesh, and then washed with deionized water and acetone before use. The as-prepared EP-Cefalexin@MSNs coating was painted on the 304SS substrate by rolling and dried at 40 °C for 12 h in vacuum. The roller (OSP-06/60) has a diameter of 10 mm and gives a dry coating thickness of 3 ± 1 µm.

### 2.4. Characterization

High resolution transmission electron micrographs (HRTEM) were carried out on a FEI Tecnai G2 F20 field emission transmission electron microscope (Hillsboro, OR, USA) at 200 kV. The Brunauer–Emmett–Teller (BET) and Barrett–Joyner–Halenda (BJH) methods were applied to analyze the surface area, pore size, and pore volume by N2 adsorption-desorption isotherms at −196 °C (BELSORP max-II, Osaka, Japan), respectively. Fourier transform infrared (FTIR) spectroscopy was conducted on a Niolet 6700 FTIR spectrometer instrument (Thermo Fisher Scientific, Waltham, MA, USA) using a KBr pellet. The UV–Vis spectra were obtained on a Mapada UV-1800 spectrometer (Mapada, Shanghai, China) to test the release performance of the Cefalexin@MSNs. Cefalexin aqueous solutions with concentrations of 0.6, 1.0, 1.4, 1.8, 2.2, 2.6, and 3.0 mg/mL were prepared, and the corresponding absorbance (A) was measured at 670 nm using the reagent blank as reference. A regression equation between A and concentration C (mg/mL) was obtained

(1) C=7.52A−0.4419

### 2.5. Electrochemical Measurements

A conventional three-electrode system was applied to conduct the electrochemical measurements on an electrochemical workstation (AUTOLAB PGSTAT302N, Zofingen, Switzerland). The reference electrode was a saturated calomel electrode (SCE), the counter electrode was a platinum electrode (1 cm^2^), and the working electrode was the 304SS coated with different coatings and sealed with 1 cm^2^ exposed area. The corrosion resistance of coated 304SS was evaluated in 3.5 wt.% NaCl solution (pH = 6.81). All measurements were conducted in a Faraday cage at 25 °C to avoid external interference. Tafel curves were obtained at a rate of 10 mV/s in a potential range of −0.25 to 0.30 V (vs. SCE). Electrochemical impedance spectra (EIS) were recorded in the frequency range of 105 Hz to 10–2 Hz at an amplitude of 10 mV. Before the EIS measurement, the working electrode was soaked in the electrolyte for 1 h to reach a stable open circuit potential (EOCP). All EIS measurements were repeated three times to maintain reproducibility.

## 3. Results and Discussion

### 3.1. Characterization of MSNs and Cefalexin@MSNs

**Figure 1:**
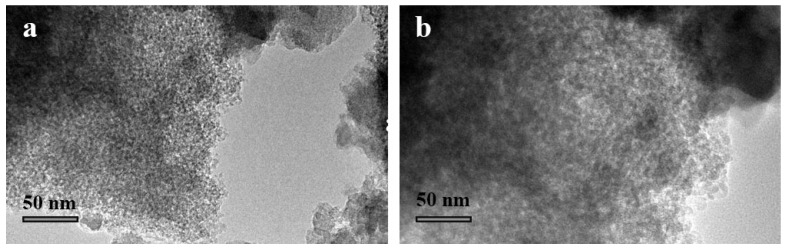
HRTEM images of Cefalexin@MSNs (**a**) and MSNs (**b**).

The TEM micrographs of the MSNs and Cefalexin@MSNs are shown in Figure 1. As observed, there are many pores in the MSNs, and the pore number decreases in the Cefalexin@MSNs, indicating the incorporation of cefalexin into the MSNs.

**Figure 2:**
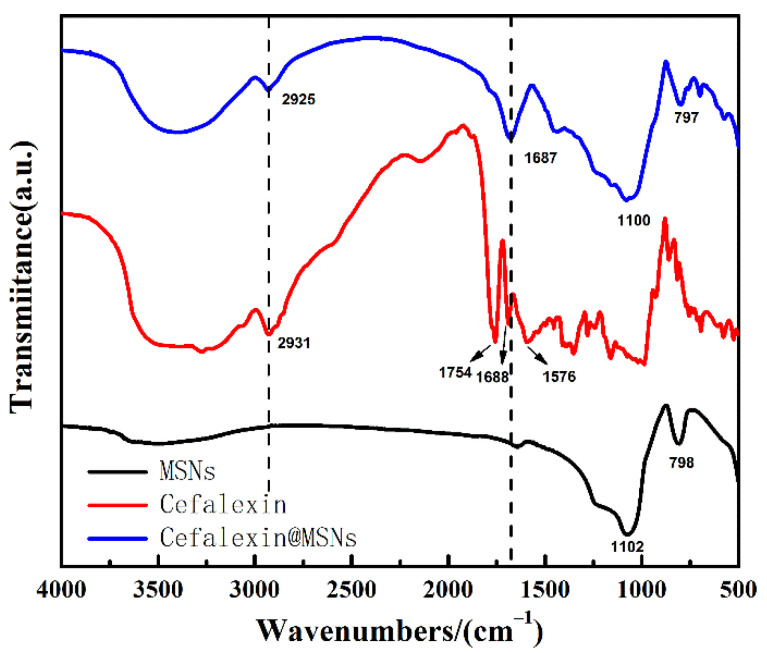
The FTIR spectra of MSNs, cefalexin, and cefalexin@MSNs.

The FTIR spectra of MSNs, cefalexin, and Cefalexin@MSNs are shown in Figure 2. The cefalexin has four obvious infrared absorption peaks at around 2925 cm^−1^,1754 cm^−1^, 1688 cm^−1^, and 1576 cm^−1^, which are ascribed to the acidic hydroxyl group in the molecule, four-membered lactam carbonyl, secondary amide carbonyl groups, and N-H bending vibrations, respectively. The peaks at 797 cm^−1^ and 1100 cm^−1^ are assigned to the typical Si-O-Si stretching and bending vibrations [30,31], which are obvious in the MSNs and the Cefalexin@MSNs. Interestingly, the characteristic peaks of cefalexin at around 2925 cm^−1^ and 1687 cm^−1^ are strong in the Cefalexin@MSNs but are absent in the MSNs, indicating the incorporation of cefalexin into the MSNs.

**Figure 3:**
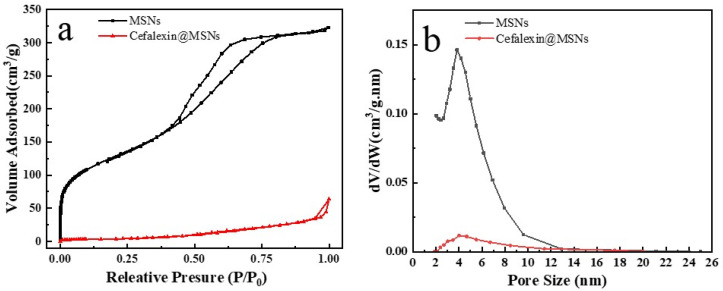
Nitrogen adsorption–desorption isotherms (**a**) and Barrett–Joyner–Halenda pore distributions (**b**) of MSNs and Cefalexin@MSNs.

The results of the nitrogen adsorption–desorption isotherms of MSNs and Cefalexin@MSNs are shown in Figure 3. The adsorption isotherm is recognized as a representative type-IV isotherms, and an obvious capillary condensation step and hysteresis loop can be observed around the relative pressure (P/P_0_) between 0.25 and 0.8, indicating mesoporous structure and wide pore size distribution (Figure 3a). However, there is no obvious change in the average pore diameter around at ~4 nm for either MSNs or Cefalexin@MSNs, as shown in Figure 3b. As summarized in Table 2, the BET surface area, the total pore volume, and the average pore diameter of the MSNs are 593 cm^2^ g^−1^, 0.64 cm^3^ g^−1^, and 4.1 nm, respectively. However, these parameters greatly decrease to 28 cm^2^ g^−1^, 0.03 cm^3^ g^−1^, and 3.9 nm, respectively, for Cefalexin@MSNs, indicating that the cefalexin has been successfully incorporated into the pores of MSNs.

### 3.2. Slow Release of Cefalexin@MSNs

**Figure 4:**
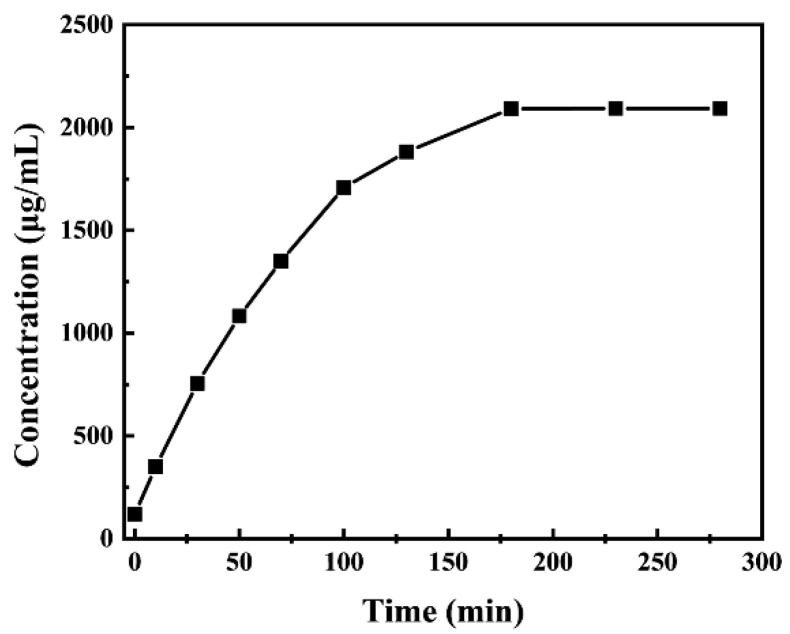
Slow-release process of Cefalexin@MSNs.

The slow-release curve of the Cefalexin@MSNs is drawn according to Formula (1). It can be seen from Figure 4 that the concentration of cefalexin in deionized water continues to increase with the increase in time. In the first 100 min, the release rate of cefalexin is fast. Over the range of 100–180 min, the release rate decreases. After 180 min, concentration remains basically unchanged, indicating that all the cefalexin loaded in the MSNs has been released. It can be seen that the slow release of cefalexin is achieved by loading the cefalexin into the mesoporous material MSNs.

### 3.3. Corrosion Resistance

**Figure 5:**
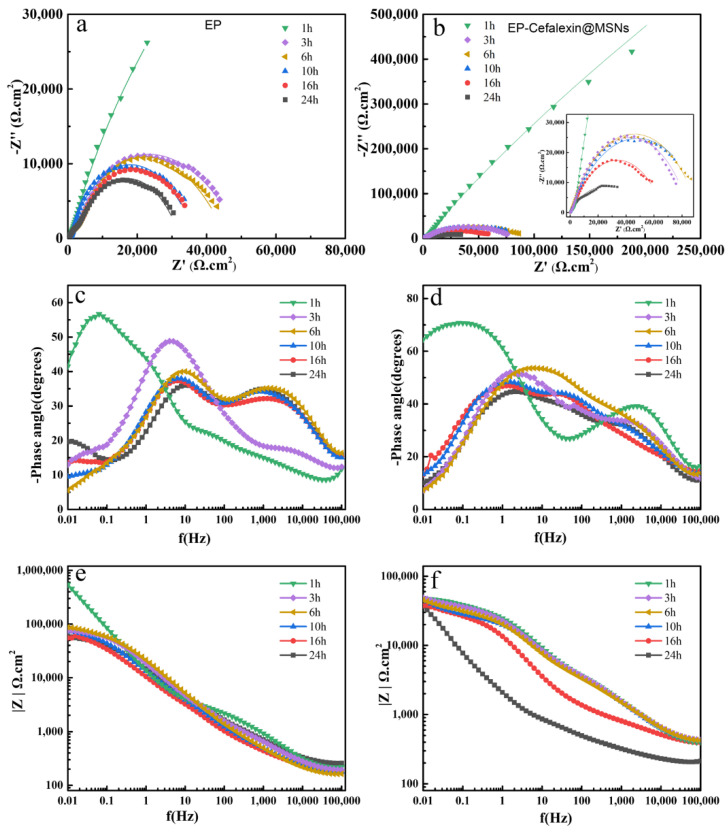
Nyquist and Bode plots of EP (**a**,**c**,**e**) and EP-Cefalexin@MSNs (**b**,**d**,**f**) after being immersed in 3.5 wt% NaCl solution for different durations.

**Figure 6:**
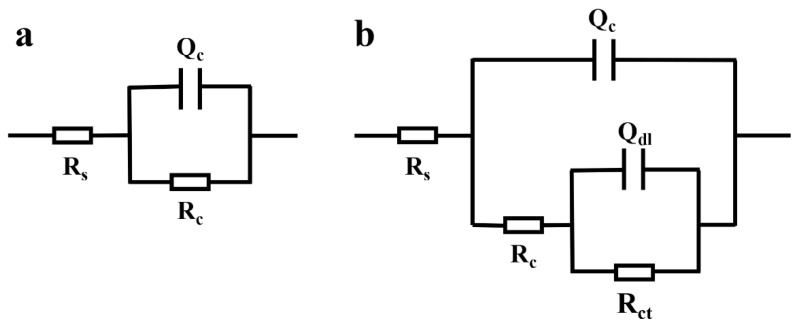
Equivalent circuit models used to fit the EIS curves, (**a**) one time constant, (**b**) two time constants.

The typical Nyquist and Bode plots of pure EP and EP-Cefalexin@MSNs in 3.5 wt.% NaCl solutions under different immersion time was shown in Figure 5. Generally, the impedance at the low frequency (|Z|0.01Hz) could measure the overall barrier performance of the coating, and the higher value of |Z|0.01Hz indicates better anticorrosive property. Figure 5a,b is the Nyquist diagrams of EP and EP-Cefalexin@MSNs coatings. Both EP and EP-Cefalexin@MSNs coatings show the maximum diameter semicircles with the highest coating resistance (Table 3) after 1 h immersion, implying good barrier effects. However, the diameter of the capacitive loop reduces with the immersion time, accompanying a decrease in charge transfer resistance (Table 3), indicating degradation of the coatings. It can be seen that the EP-Cefalexin@MSNs coating has a larger diameter than EP coating during 24 h immersion, suggesting its better corrosion resistance. Figure 5c,d is the Bode diagrams of EP and EP-Cefalexin@MSNs coatings. The high-frequency Bode phase angle plot deals with the local coating defects and solution resistance, and the low frequency region is related to the electrolyte/coating interface [32]. The phase angles at high frequencies for the EP-Cefalexin@MSNs coating are more negative than those for the EP, indicating a good barrier for SS. As shown in Figure 5e,f, the value of |Z|_0.01Hz_ of EP coating decreases with the increase in immersion time. For the EP-Cefalexin@MSNs coating, the value of |Z|_0.01Hz_ is one order of magnitude greater than EP coating after being immersed for 1 h. After being immersed for 24 h, the value decreased to 54,907 Ω cm^2^, which is still higher than that of EP coating (36,884 Ω cm^2^), demonstrating a better barrier performance of cefalexin@MSNs coating. In order to obtain quantitative information from the EIS plot, the equivalent circuits in Figure 6 are proposed to fit EIS data. CPE (Q) is used as a substitute for ideal capacitive element (C) for a better fit of depressed capacitive loops. R_s_, R_c_, and R_ct_ represent solution resistance, coating resistance, and charge transfer resistance, respectively. A constant phase element (CPE) is used to describe the nonideal capacitive response of the corrosion system. Q_c_ and Q_dl_ are CPEs related to the coating capacitance (C_c_) of the coating/electrolyte interface and double layer capacitance (C_dl_) of the metal/electrolyte interface, respectively. The real capacitance (C) including CPE is deduced by the following equation [33].

$$C=Q{\left(\omega_{\max}\right)}^{n-1}$$

where Q is the CPE constant and ω_max_ is the angular frequency at which the Z imaginary is maximum. When n = 1, CPE behaves like an ideal capacitor, whereas when n = 0, CPE denotes an ideal resistor. The fitted values of EIS parameters are shown in Table 3. The larger the R_c_ and the smaller the C_c_, the better the corrosion resistance of the coating [34,35,36]. It can be seen from Figure 5 and Table 3 that the R_c_ value of pure epoxy resin is largest at the initial stage of immersion, which indicates that the EP coating acts as a physical barrier. As the immersion time increases, corrosive substances slowly penetrate into the coating, and the value of R_c_ begins to gradually decrease. Similarly, the charge transfer resistance (R_ct_) value of pure EP dropped to 29,476 Ω cm^2^ after 16 h immersion, implying that the effect of the physical barrier was weakened. With the immersion time increases to 24 h, R_c_ drops to 991 Ω cm^2^, reflecting the continuous degradation of coating after long-term immersion.

The results show that the EP-Cefalexin@MSNs coating and the pure EP coating have similar properties but have significant differences. The R_c_ value of the EP-Cefalexin@MSNs coating at the initial stage of immersion is much greater than that of pure EP. This is because the added MSNs increase the physical barrier effect of the coating. In addition, the R_ct_ value for 6 h (90,659 Ω cm^2^) immersion is higher than that for 3 h (88,067 Ω cm^2^) immersion. During the 3–10 h of immersion, the rate of change of the R_ct_ value of the EP-Cefalexin@MSNs coating is very slow. This is attributed to the release of cephalexin from the MSNs as the increase of immersion time, inhibiting the corrosion of SS. When immersed for 24 h, the R_ct_ value of the EP-Cefalexin@MSNs coating decreases to 58,961 Ω cm^2^ but is still much higher than that of the EP coating, which indicates that EP-Cefalexin@MSNs has more long-term corrosion resistance.

**Figure 7:**
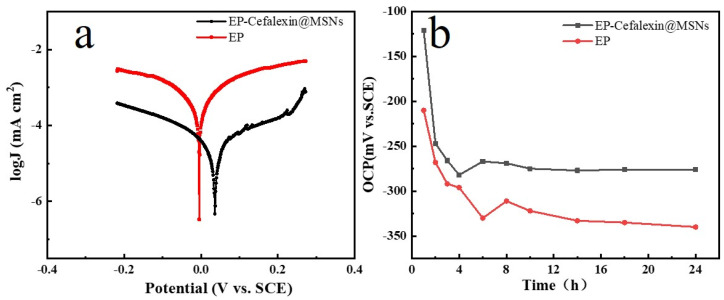
Tafel curves (**a**) and OCP (**b**) of EP and EP-Cefalexin@MSNs in 3.5 wt.% NaCl solution.

It can be seen from Figure 7a and Table 4 that both the anodic and cathodic Tafel curves shift to the low current densities and that the corrosion potentials also move to the positive direction. The current density (I_corr_) decreases from 0.5236 μA cm^−2^ in EP coating to 0.04275 μA cm^−2^ in EP-Cefalexin@MSNs coating, indicating that the anodic corrosion of carbon steel is suppressed gradually due to the released cefalexin from Cefalexin@MSNs. In addition, the I_corr_ of some related EP-based coatings on 304 SS in 3.5 wt.% NaCl solution was provided in Table 5, which shows that in our work, the EP-Cefalexin@MSNs have a lower I_corr_ than the other prior systems, indicating better corrosion resistance. It can be seen from Figure 7b that the OCP of the EP-Cefalexin@ MSNs coating is higher than that of the EP coating, demonstrating that the corrosion resistance of the EP-Cefalexin@MSNs coating is better than that of the EP coating.

### 3.4. Self-Healing Mechanism

**Figure 8:**
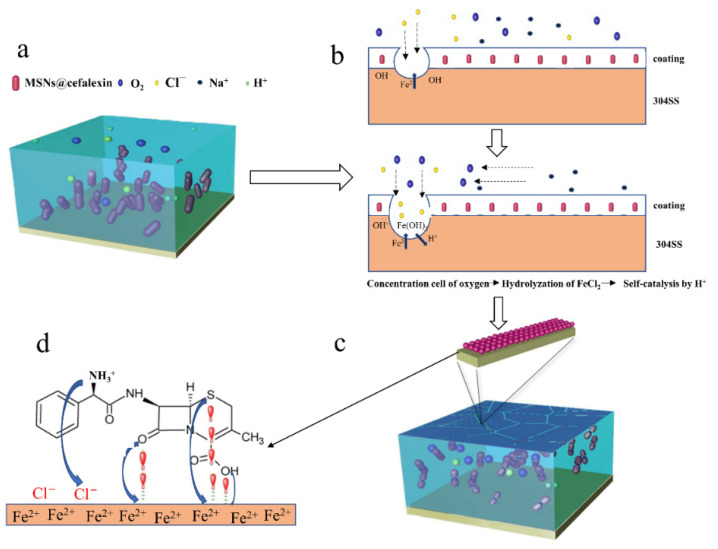
The self-healing mechanism of EP-Cefalexin@MSNs coating: (**a**) Before coating corrosion, (**b**) corrosive medium penetrates into the coating, (**c**) the self-healing process, (**d**) adsorption principle of Cefalexin molecular.

Figure 8 illustrates the self-healing mechanism of the EP-Cefalexin@MSNs coating. When defects such as pores and microcracks appear in EP-Cefalexin@MSNs coating after immersion in NaCl solution, the following electrochemical corrosion reactions occur in these defects.

The positive polar reactions:

(2) $$\mathrm{Fe}-2e\to {\mathrm{Fe}}^{2+}$$

(3) $${\mathrm{Fe}}^{2+}+2{\mathrm{Cl}}^{-}\to {\mathrm{FeCl}}_{2}$$

(4) $${\mathrm{FeCl}}_{2}+2H_{2}O\to \mathrm{Fe}{\left(\mathrm{OH}\right)}_{2}+2\mathrm{HCl}$$

The negative polar reaction:

(5) $$2H^{2+}+2e^{-}\to H_{2}$$

Owing to their small size and strong penetration ability, chloride ions tend to squeeze out oxygen atoms in the passivation film, leading to the formation of FeCl_2_. The FeCl_2_ hydrolyzes and produces HCl, accelerating the SS’s corrosion by the self-catalysis in this area (Figure 8b). In an acidic environment, the amino groups of the cefalexin are protonated and positively charged and are adsorbed on the 304SS by electrostatic attraction due to the negatively charged chloride ions [28]. Fe in the 304SS has a greater affinity to coordinate with ligands bearing unshared electron pairs of heteroatoms and hence forms the protective film [40,41]. The cefalexin released from the EP-Cefalexin@MSNs adsorbs on the 304SS through unshared electron pairs in O and S, leading to the formation a protection layer (Figure 8d). In addition, with the increase in the adsorbed cefalexin on SS, the double electric layer around the SS’s surface is compressed through intermolecular hydrogen bonds, resulting in an increase in capacitance and further inhibition of corrosion.

## 4. Conclusions

This work creatively prepared a self-healing EP coating by employing the expired cefalexin loaded into the mesoporous silica nanomaterials as corrosion inhibitor. The self-healing EP coating can intelligently release cefalexin when corrosion occurs, which adsorbs on the surface of the 304 SS to protect it from corrosion. Compared with the pure EP coating, the EP-cefalexin@MSNs coating has a higher self-corrosion voltage, a lower self-corrosion current density, and 2 times higher R_ct_ after 24 h immersion. The improved corrosion resistance is attributed to the densified adsorption of the cefalexin inhibiting the cathode corrosion reaction, providing a self-healing and long-duration corrosion protection for 304SS.

## Figures and Tables

**Table 1:** Composition of 304SS/wt.%.

Cr	Mn	Si	Ni	Mo	C	P	S	Fe
18.50	0.88	0.59	8.12	0.30	0.05	0.015	0.028	Balance

**Table 2:** S_BET_, V_p_, and D_BJH_ of MSNs and Cefalexin@MSNs.

Sample	S_BET_ (m^2^ g^−1^)	Vp (cm^3^ g^−1^)	D_BJH_ (nm)
Pure-MSNs	593	0.64	4.1
Cefalexin-MSNs	28	0.03	3.9

**Table 3:** The EIS fitting parameters for the different coatings after immersed in 3.5 wt% NaCl solution for different durations.

Sample	Time(h)	R_c_(Ω cm^2^)	Q_c_		Q_dl_		R_ct_(Ω cm^2^)
			C_c_(F/cm^2^)	n	C_dl_(F/cm^2^)	n	
EP	1	262,880	5.42 × 10^−7^	0.7235	-	-	-
	3	6662	3.08 × 10^−6^	0.7522	4.05 × 10^−6^	0.6358	35,398
	6	6394	4.94 × 10^−6^	0.7633	4.29 × 10^−6^	0.7756	34,164
	10	5971	5.17 × 10^−6^	0.7521	4.44 × 10^−6^	0.7855	33,665
	16	5396	6.02 × 10^−6^	0.7825	6.63 × 10^−6^	0.7542	29,476
	24	991	8.70 × 10^−6^	0.7963	9.68 × 10^−6^	0.7865	23,649
EP-Cefalexin@ MSNs	1	542,235	4.52 × 10^−7^	0.7562	-	-	-
	3	99,685	2.44 × 10^−6^	0.769	3.42 × 10^−6^	0.7564	88,067
	6	100,675	2.05 × 10^−6^	0.7848	3.12 × 10^−6^	0.7756	90,659
	10	99,002	2.55 × 10^−6^	0.7935	3.50 × 10^−6^	0.7867	88,652
	16	81,003	3.59 × 10^−6^	0.7252	4.52 ×10^−6^	0.7683	64,562
	24	70,236	4.95 × 10^−6^	0.7741	5.30 × 10^−6^	0.7848	58,961

**Table 4:** The kinetic parameters of the Tafel curves.

Sample	E_corr_/mV VS. SCE	I_corr_/μA/cm^2^
EP	−19	0.5236
EP-Cefalexin@ MSNs	58	0.04275

**Table 5:** The Icorr of different coatings on 304 SS in 3.5 wt.% NaCl solution (μA/cm^2^).

Systems	Icorr (Samples)	
Ref [37]	0.71 (EP)	0.25 (EP-PANI)
Ref [38]	0.46 (EP)	0.06 (EP-graphene)
Ref [39]	1.82 (EP)	0.615 (EP-SnO_2_)
Our work	0.5236 (EP)	0.04275 (EP-Cefalexin@ MSNs)

## Data Availability

Not applicable.
